# Exploring the mechanism of Zishen Quyu Jiedu formula in treating endometriosis based on network pharmacology and experimental verification

**DOI:** 10.3389/fendo.2025.1667486

**Published:** 2025-10-28

**Authors:** Ruolin Wang, Xinyang Tian, Peiyu Liu, Fang Lian

**Affiliations:** ^1^ First Clinical Medical College, Shandong University of Traditional Chinese Medicine, Jinan, Shandong, China; ^2^ Institute of Traditional Chinese Medicine Literature and Culture, Shandong University of Traditional Chinese Medicine, Jinan, Shandong, China; ^3^ State Key Laboratory of Traditional Chinese Medicine Syndrome/The Second Clinical College, Guangzhou University of Chinese Medicine, Guangdong, China

**Keywords:** oxidative stress, endometriosis, blood stasis with toxin accumulation, ZSQYJDF, nuclear factor E2-related factor 2

## Abstract

**Objective:**

Zishen Quyu Jiedu formula (ZQYJDF) is a commonly used prescription for endometriosis (EMs) with clinical efficacy. However, its active components and potential mechanisms remain unknown. This study aimed to identify the key targets and signaling pathways involved in the treatment of EMs by ZSQYJDF and to clarify its mechanism.

**Materials and methods:**

Multiple databases were integrated to screen the effective components of ZSQYJDF and their protein targets, with redundancies removed. EMs-related genes were obtained from several disease databases. A drug–component–target network was constructed using overlapping targets. Gene Ontology (GO) and Kyoto Encyclopedia of Genes and Genomes (KEGG) enrichment analyses were performed to identify key pathways and proteins. An autologous transplantation rat model of EMs was established. Hematoxylin and eosin (H&E) staining was used to observe lesion morphology; immunohistochemistry (IHC) was used to assess positive expression of NRF2, HO1, and NQO1; serum SOD, MDA, GSHPx, 8epiPGF2α, IL6, IL1β, and TNFα were measured by ELISA; and mRNA and protein levels of NRF2, HO1, NQO1, and KEAP1 in endometrial tissue were detected by qPCR and Western blot.

**Results:**

comparisonon of the screened compounds with 1,225 known disease-related targets identified 134 potential targets for ZSQYJDF. GO terms were enriched in response to oxidative stress and cellular responses to oxidative stress. KEGG pathways were enriched in the TNF, NRF2, and HIF signaling pathways. HPLC-QOrbitrap-MS identified and inferred 48 compounds. In *in vivo* experiments,ZQYJDF reduced inflammatory cell infiltration in ectopic endometrial stroma, leading to local atrophy of lesions, decreased IL-6, IL-1β, TNF-α, 8-epi-PGF2α, and MDA, increased the expression of NRF2, NQO1, and HO1, and decreased KEAP1.

**Conclusion:**

Utilizing methods including network pharmacology, HPLC-Q-Orbitrap-MS component identification, and animal experiments, the main active components and potential therapeutic targets of ZSQYJDF were identified, and its mechanism of action in treating EMs was preliminarily elucidated, providing a scientific basis for further research on EMs.

## 1 Introduction

Endometriosis (EMs) is a common gynecological disorder characterized by the growth of endometrium-like tissue outside the uterine cavity (1). In recent years, its global incidence has continued to rise. An estimated 10% of women of reproductive age are affected, with more than 60 million patients in China, and a marked increase has been observed in younger women (2). EMs is not only a localized pelvic condition but also a systemic chronic inflammatory disease with complex and diverse etiology. Ectopic lesions exhibit features resembling malignancy, including proliferation, adhesion, invasion, and migration, accompanied by estrogen-driven chronic inflammation. The disease involves extensive and heterogeneous lesions that seriously impair physiological function and quality of life, with clinical manifestations such as chronic pelvic pain, dysmenorrhea, dyspareunia, menstrual disorders and infertility. Due to its invasiveness and high recurrence rate, EMs is often regarded as a “benign cancer,” posing a substantial challenge to women’s health worldwide (3). The exact pathogenesis remains unclear. Although the retrograde menstruation theory is prevalent, it does not explain all clinical phenomena (4). Current treatments mainly suppress estrogen production and include gonadotropin-releasing hormone agonists (GnRHa), progestins, and surgery (5). Therefore, new drugs and therapeutic strategies are urgently needed.

Substantial evidence indicates that Oxidative Stress (OS)and inflammation play central roles in the onset and progression of EMs, mutually reinforcing each other and forming a vicious cycle (6). In an abnormal peritoneal environment, ectopic endometrial cells are metabolically active and generate excessive Reactive Oxygen Species (ROS), leading to elevated local OS (7). Excess ROS not only causes direct cellular damage but also acts as signaling molecules to activate inflammatory pathways, such as nuclear factor kappa B (NF-κB), promoting the release of pro-inflammatory cytokines, including tumor necrosis factor-α (TNF-α) and interleukin-6 (IL-6). This exacerbates local inflammation and facilitates angiogenesis and cell proliferation. In turn, persistent inflammation enhances OS, driving disease progression (8). Nuclear factor erythroid 2-related factor 2 (NRF2) is a central regulator of the cellular antioxidant response and a key node for disrupting this vicious cycle. Activation of the NRF2 pathway upregulates antioxidant and phase II detoxification enzymes, such as heme oxygenase-1 (HO-1) and NAD(P)H quinone dehydrogenase 1 (NQO1), effectively eliminating ROS and exerting anti-inflammatory effects through negative regulation of NF-κB and related pathways (9). Thus, targeting NRF2 activation to counter OS and inflammation simultaneously represents a promising strategy for treating EMs.

Traditional Chinese medicine (TCM) is a distinct medical system (10, 11). TCM treatments do not suppress the pituitary–ovarian axis or interfere with the normal menstrual cycle (12, 13). In recent years, TCM formulas have shown significant efficacy in the clinical prevention and treatment of the onset and progression of EMs (14–17). They also exhibit obvious efficacy in relieving pain and discomfort, controlling the progression of lesions, and regulating menstruation. Moreover, TCM has fewer side effects, making it suitable for treating chronic non-severe conditions. In TCM theory, the pathogenesis of EMs involves blood stasis, in which blood that “departs from the meridian” accumulates ectopically, often lodging in the lower abdomen and obstructing the Chong-Ren channels and uterus. ZSQYJDF (18), developed by Professor Lian Fang based on extensive clinical experience, comprises daxueteng;Meiguihua;Jinyinhua;Lianqiao; Danggui; Baishao;Shengdihuang; Zhigancao;Chuanxiong;Danshen; Tusuzi;Gouqizi.This formula has complex constituents and acts on multiple targets and pathways. Clinical studies have reported the beneficial effects of ZSQYJDF on EMs, including inhibition of lesion growth, improvement of serum CA125 levels, relief of blood stasis with toxin accumulation symptoms, increased fertilization rate, number of embryos, and high-quality embryo rate (19–22). However, its pharmacologically active components and molecular mechanisms require further elucidation.

Based on this background, we hypothesized that ZSQYJDF could treat EMs by activating the NRF2 signaling pathway, thereby improving OS and inflammatory responses. To verify this hypothesis, we for the first time integrated a multidimensional research strategy incorporating network pharmacology prediction (23), literature analysis, HPLC-Q-Orbitrap-MS component identification (24–27), and *in vivo* experimental validation. The potential active components, therapeutic targets, and signaling pathways of this formula were screened using network pharmacology. An EMs rat model was established using the autotransplantation method to evaluate the therapeutic effects at the whole-animal level. The mechanism of action of this combination therapy was intensively investigated, focusing on OS, inflammatory factor levels, and protein expression of the NRF2/HO-1/NQO1 pathway. This study aimed to elucidate the effective components and mechanisms of action of ZSQYJDF in the treatment of EMs, thereby providing a new theoretical and experimental basis for its application in the treatment of EMs.

## 2 Materials and methods

### 2.1 Network pharmacology

#### 2.1.1 Collection of active components and targets of ZSQYJDF

Active components were screened from the TCMSP database using the herb list: Danshen, Da Xue Teng, Jinyinhua, Lianqiao, Meiguihua, Chuanxiong, Chao Baishao, Sheng Dihuang, Danggui, and Zhi Gancao (**Table 1**). The screening criteria were oral bioavailability (OB) ≥ 30% and drug-likeness (DL) ≥ 0.18. The active components of the ZSQYJDF were obtained. The chemical structures and simplified molecular-input line-entry system (SMILES) strings of these components were retrieved from the TCMSP database, PubChem (https://pubchem.ncbi.nlm.nih.gov/), and the related literature in CNKI. The obtained structures were uploaded to the Swiss Target Prediction (STP) and SuperPred databases, with species set to Homo sapiens and a probability threshold of > 0. The predicted targets were standardized using the UniProt database (https://www.uniprot.org/). After removing duplicates, the final potential target genes of the components were identified.

**Table 1:** Composition of Zishen Quyu Jiedu formula.

Latin name	Chinese name	Medicinal part	Batch number	Daily adultdose (g)
Sargentodoxae Caulis	Daxueteng	Dried vine stems	Z20010123	15g
Rose Rugosae Flos	Meiguihua	Dried flower buds	Z20210001	15g
Lonicerae Japonicae Flos	Jinyinhua	Dried flower buds	Z20210010	30g
Forsythiae Fructus	Lianqiao	Dried fruits	Z20055415	15g
Radix Salviae	Danshen	Dried rhizome	Z44023372	15g
Angelicae Sinensis Radix	Danggui	Dried rhizome	Z41021479	12g
Paeoniae Radix Alba	Baishao	Dried rhizome	Z20054521	15g
Chuanxiong Rhizoma	Chuangxiong	Dried rhizome	Z20080345	12g
Rehmannia glutinosa	Shengdi	Dried tubers and roots	6931722604013	12g
Glycyrrhizae	Zhigancao	Roots and rhizomes	Z20050432	6g
Cuscutae Semen	Tusizi	mature seed	Z20180225	15g
Babury Wolfberry Fruit	Gouqizi	ripening fruits	Z20203228	15g

#### 2.1.2 Potential targets for EMs

EMs-related targets were collected by searching with the keyword “endometriosis.” In GeneCards (https://www.genecards.org/), targets with scores > 0.9 were selected for analysis. Similarly, EM-related targets were retrieved from OMIM (https://www.omim.org/) and DisGeNET (https://www.disgenet.org). The UniProtKB function in UniProt (https://www.uniprot.org/) was used to calibrate the protein targets, and duplicates were removed to obtain EMs-related targets. A multilayer network of drug–active component–target interactions was constructed.

#### 2.1.3 Network construction of ZSQYJDF – active components – targets

The targets corresponding to ZSQYJDF and EMs active components were uploaded to Venny 2.1.0 to draw a Venn diagram; the intersecting genes represented predicted targets related to the disease. These intersecting targets, together with ZSQYJDF–associated active components, were visualized as a multi-layer network of “ZSQYJDF – active components – targets” in Cytoscape 3.7.1.

#### 2.1.4 Protein–protein interaction network of disease targets

The intersecting targets were submitted to the STRING online platform with a confidence score cutoff of > 0.90 (high confidence) to obtain the PPI network. The resulting PPI data were imported into Cytoscape (version 3.7.1). Using the BiNGO/BIOSIG-style workflow via the BiNGer/CytoNCA plugins, we analyzed topological parameters, including Degree, Betweenness Centrality, and Closeness Centrality, to identify core targets and further refine key bioactive compounds and core targets.

#### 2.1.5 GO and KEGG pathway enrichment analyses

The intersecting gene set was subjected to GO and KEGG enrichment analyses using DAVID (https://david.ncifcrf.gov/). Term selection followed the default settings, with the species set to Homo sapiens. The results were visualized using R as bubble plots.

### 2.2 HPLC–QOrbitrapMS analysis

Sample preparation and LC–MS analysis were performed as follows: thawed samples were vortexed for 30 s to mix thoroughly, then centrifuged at 12,000 rpm for 10min at 4°C. A 200 µL aliquot of the supernatant was transferred to a 1.5 mL microcentrifuge tube, and 1,000 µL methanol–water (4:1, v/v) extraction solvent was added, vortex-mixed for 10min, and centrifuged again at 12,000 rpm for 10min at 4°C. The collected supernatant was filtered through a 0.22 µm organic membrane filter and transferred to HPLC auto-sampler vials for analysis.

Chromatographic separation was performed using a Welch AQC18 column (1.8 µm, 150 × 2.1mm). The mobile phase consisted of 0.1% formic acid in water (A) and methanol (B). The flow rate was 300 µL/min, and the injection volume was 5 µL (**Table 2**).

The gradient program was as follows: 0–5 min, B from 2% to 20%; 5–10 min, to 50%; 10–15 min, to 80%; 15–20 min, to 95%; hold to 27min.

The mass spectrometric conditions were as follows: sheath gas, 40 arb; auxiliary gas, 15 arb; positive ion mode; spray voltage, 3.2 kV; and capillary temperature, 300°C. Acquisition parameters: full scan resolution 70,000; MS/MS resolution 17,500; normalized collision energy (NCE) 30%.

Chemical component analysis of the samples was performed by Wuhan Saiweier Biotechnology Co., Ltd.

### 2.3 Animal experimental validation

#### 2.3.1 Experimental animals

24 SPF-grade, healthy, nulliparous female Sprague–Dawley (SD) rats, 8 weeks of age and weighing200 ± 10g, were purchased from Jinan Pengyue Laboratory Animal Breeding Co., Ltd. [production license: SCXK (Lu) 2019-0003]. Animals were housed at the Animal Experiment Center of Shandong University of Traditional Chinese Medicine (Jinan, Shandong, China). All rats were maintained under strictly controlled and identical environmental conditions: temperature 23–24°C, relative humidity 50–60%, and a 12h light/dark cycle. General health was monitored daily, and body weight and food intake were measured and recorded on a weekly basis. The dose was calculated and adjusted weekly according to body weight. Animal management and procedures were consistent across all groups. All procedures were approved by the Animal Ethics Committee of Shandong University of Traditional Chinese Medicine (Approval No. SDSZYYAWE20240710001).

#### 2.3.2 Preparation of drugs

##### 2.3.2.1 ZQYJD Fcomposition

**Figure 1:**
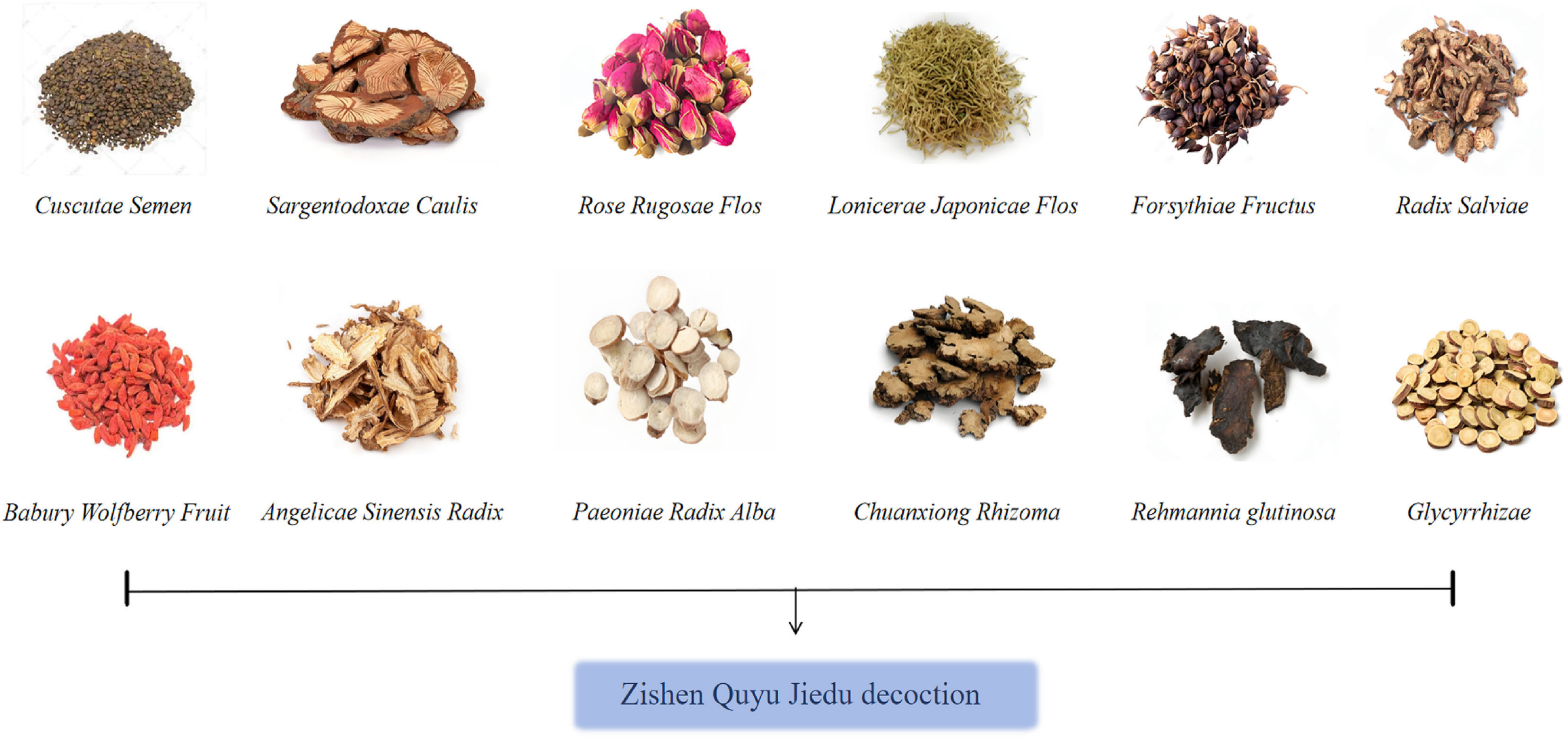
Traditional Chinese Medicines in the Zishen Quyu Jiedu Formula and Their Names.

Danshen 15g,Da Xue Teng 15g,Jinyinhua30 g, Lianqiao 15g, Meiguihua15 g, Chuanxiong 12g, Baishao 15g, Sheng Dihuang12 g, Danggui 12g,Tusizi15g,Gouqizi15g,Zhi Gancao 6g (**Figure 1**, **Table 2**). The decoction pieces were sourced from the Affiliated Hospital of Shandong University of Traditional Chinese Medicine. The herbs were mixed, an appropriate volume of distilled water was added, decocted for 60min, and then filtered. Twice the volume of distilled water was added to the dregs, and the mixture was decocted for 40min and filtered. The two filtrates were combined and gently concentrated to obtain an aqueous decoction at 4.802 g/mL, which was stored at 4°C until use.

**Table 2:** Chromatographic gradient.

Time(min)	Water phase ratio(%)	Organic phase ratio(%)
0.0	98	2
1	98	2
5	80	20
10	50	50
15	20	80
20	5	95
25	5	95
26	98	2
30	98	2

##### 2.3.2.2 Mifepristone (China Resources Zizhu Pharmaceutical Co., Ltd., Specification

25 mg, Batch No.: C16320-04-0).Preparation: Mifepristone was suspended in double-distilled water and mixed to obtain a 0.125 mg/mL mifepristone suspension.

#### 2.3.3 Establishment of the rat EMs model and dosing

**Figure 2:**
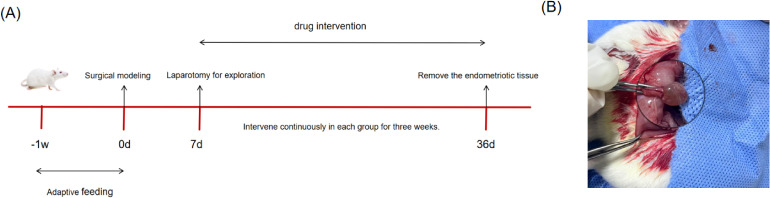
**(A)** Animal feeding flowchart. **(B)** Schematic diagram of successful modeling of rat EMs by autotransplantation. The transplanted volume increased, with translucent nodules or cystic vesicles and neovascularization at the transplantation site.

24 female SD rats were acclimatized for 7 days and randomly assigned to four groups—blank, model,ZSQYJDF, and mifepristone (n = 6)–to avoid selection bias. An EMs model was established using autologous transplantation (28). Under anesthesia, a laparotomy was performed, and one uterine horn was ligated at both ends and excised. The uterine tissue was opened longitudinally and divided into two segments. The endometrial surface of each segment was sutured to the bilateral abdominal wall, and the abdomen was closed. Penicillin was administered postoperatively for infection prophylaxis. Ten days later, the abdomen was reopened to assess ectopic lesion growth. Macroscopically, the grafts appeared enlarged and raised, with translucent nodules or cystic vesicles and neovascularization at the implantation site, indicating successful modeling (**Figure 2B**).

Dosing began the next day. Dose levels were calculated according to a human–animal body surface area conversion table (29). The ZSQYJDF and mifepristone groups received ZSQYJDF (40 g/kg) and mifepristone (0.02 g/kg) by gavage. The blank and model groups received equal volumes of normal saline via gavage. After 21 consecutive days, ectopic endometrial tissue was collected (**Figure 2A**, **Table 3**).

**Table 3:** Summary of administration after model establishment in animals.

Group	Drug name	Drug administration dose	Drug administration cycle
Blank Control	Normal Saline	–	21 days
Model	Normal Saline	–	21 days
ZSQYJDF	Zishen Quyu Jiedu formula	40g/kg	21 days
Mifepristone	Mifepristone Suspension	0.02g/kg	21 days

#### 2.3.4 H&E staining

Sections of ectopic uterine lesions from rats (normal uterine tissue from the blank control group) were fixed with paraformaldehyde, embedded in paraffin, and serially sectioned coronally at 5-µm intervals., embedded in paraffin, and sectioned at 5 µm at regular intervals. The sections were stained with hematoxylin and eosin (H&E) at room temperature (hematoxylin for 3min, eosin for 15 s), followed by dehydration, clearing, and mounting. Blinded histological assessment was performed under a light microscope at 200× magnification to evaluate endometrial epithelium thinning/atrophy, reduction and shrinkage of stromal cells, compactness of cellular arrangement, and glandular atrophy. Representative images were obtained.

#### 2.3.5 Immunohistochemical staining and evaluation criteria

The expression of Nrf2, HO-1, and NQO1 in rat endometrial tissue was detected using immunohistochemistry. Endometrial tissues fixed in 4% paraformaldehyde were dehydrated, cleared, infiltrated with wax, embedded, sectioned, and baked. Suitable sections were selected, dewaxed, rehydrated, and subjected to antigen-retrieval. Primary antibodies (diluted 300-fold) were applied and incubated overnight at 4°C. After washing, HRP-conjugated secondary antibodies (diluted 1000-fold) were added and incubated at room temperature for 30min. Freshly prepared DAB solution was applied for color development at room temperature for 10min, and the reaction was immediately stopped by placing the slides in water. This was followed by counterstaining with hematoxylin for 30 s, dehydration, clearing, and mounting. Images were captured using an optical microscope, and the average optical density (OD) value was calculated and analyzed using the ImageJ software.

The immunohistochemical results were independently evaluated by two blinded pathologists using a semi-quantitative scoring system. The staining intensity was scored on a scale of 0–3 (0: no staining; 1: weak staining, light yellow; 2: moderate staining, brownish-yellow; 3: strong staining, brown). The percentage of positive cells was scored on a scale of 0–4 (0: <5%; 1: 5%–25%; 2: 26%–50%; 3: 51%–75%; 4: >75%). The H-Score (comprehensive score) for each sample was calculated as “staining intensity score × positive cell percentage score,” yielding a range of 0–12. The average score of the two evaluators was used for the subsequent statistical analysis.

#### 2.3.6 Enzymelinked immunosorbent assay

Frozen serum samples were used to determine SOD, GSHPx, MDA, 8epiPGF2α, TNFα, IL6, and IL1β by ELISA, strictly following the manufacturers’ instructions.

#### 2.3.7 Western blot

For each group, three tissue samples were homogenized in RIPA lysis buffer containing PMSF and phosphatase inhibitors and lysed on ice for 30min to extract the total protein. Protein concentration was determined by BCA assay. Equal amounts of protein (10 µg per lane) were separated on 10% SDS-PAGE gels and transferred to PVDF membranes, followed by blocking. Membranes were incubated with primary antibodies against NRF2 (1:2500), KEAP1 (1:5000), HO1 (1:3000), NQO1 (1:25000) (**Table 4**), and βactin (1:50000) overnight at 4°C, washed with TBST three times (5min each), and then incubated with secondary antibody (1:5000) for 1h. After three washes with TBST, the bands were developed using ECL reagents and imaged. Band intensity was quantified using ImageJ by grayscale densitometry.

**Table 4:** Summary of antibodies and antibody dilution ratios.

Antibody name	Manufacturer	Concentration	Batch number
β-Actin	ABclonal	1:50000ul	AC026
NRF2	Proteintech	1:2500ul	R1312-8
HO1	ABclona	1:3000ul	A23650
NQO1	Proteintech	1∶25000ul	HA721525
KEAP1	Proteintech	1∶5000ul	ET1702-50

#### 2.3.8 Quantitative real-time PCR

Three tissue samples per group were selected. Total RNA was extracted using the SPARKeasy Tissue/Cell RNA Rapid Extraction Kit and reverse-transcribed into cDNA. Reaction mixtures were prepared according to the 2× SYBR Green qPCR Mix kit instructions, including cDNA and primers for the target and reference genes (primers designed and synthesized by Sangon Biotech Co., Ltd., Shanghai; see **Table 5**) and amplified on a PCR instrument.

**Table 5:** Primer sequences.

Gene name	Forward primer	Reverse primer	Product length
NRF2	GCCTTCCTCTGCTGCCATTAGTC	TGCCTTCAGTGTGCTTCTGGTTG	235
HO1	TCGCATGAACACTCTGGAGATGAC	GTCTGTGAGGGACTCTGGTCTTTG	107
KEAP1	GCTCAACCGCTTGCTGTATGC	AATCATCCGCCACTCATTCCTCTC	131
NQO1	GTGAGAAGAGCCCTGATTGTATTGG	AAGTTCATAGCATAGAGGTCAGATTCG	153
β-ACTIN	CCCATCTATGAGGGTTACGC	TTTAATGTCACGCACGATTTC	97

The PCR program was as follows: pre-denaturation at 96°C for 4min, followed by 40 cycles of 96°C for 13 s and 60°C for 35 s. β-actin served as the reference gene. Relative mRNA expression levels of NRF2, KEAP1, HO-1, and NQO1 were calculated using the 2–ΔΔCt method.

### 2.4 Statistics and Analysis

This study employed two software packages for statistical analysis and visualization: Prism 9.1.1 and SPSS 25.0.

Data are presented as mean ± standard deviation (SD).

For normally distributed data, intergroup comparisons were performed using one-way ANOVA followed by Tukey’s *post hoc* test.

P < 0.05 was considered statistically significant.

## 3 Results

### 3.1 Network pharmacology analysis

#### 3.1.1 Collection of active components and target prediction for ZSQYJDF

**Figure 3:**
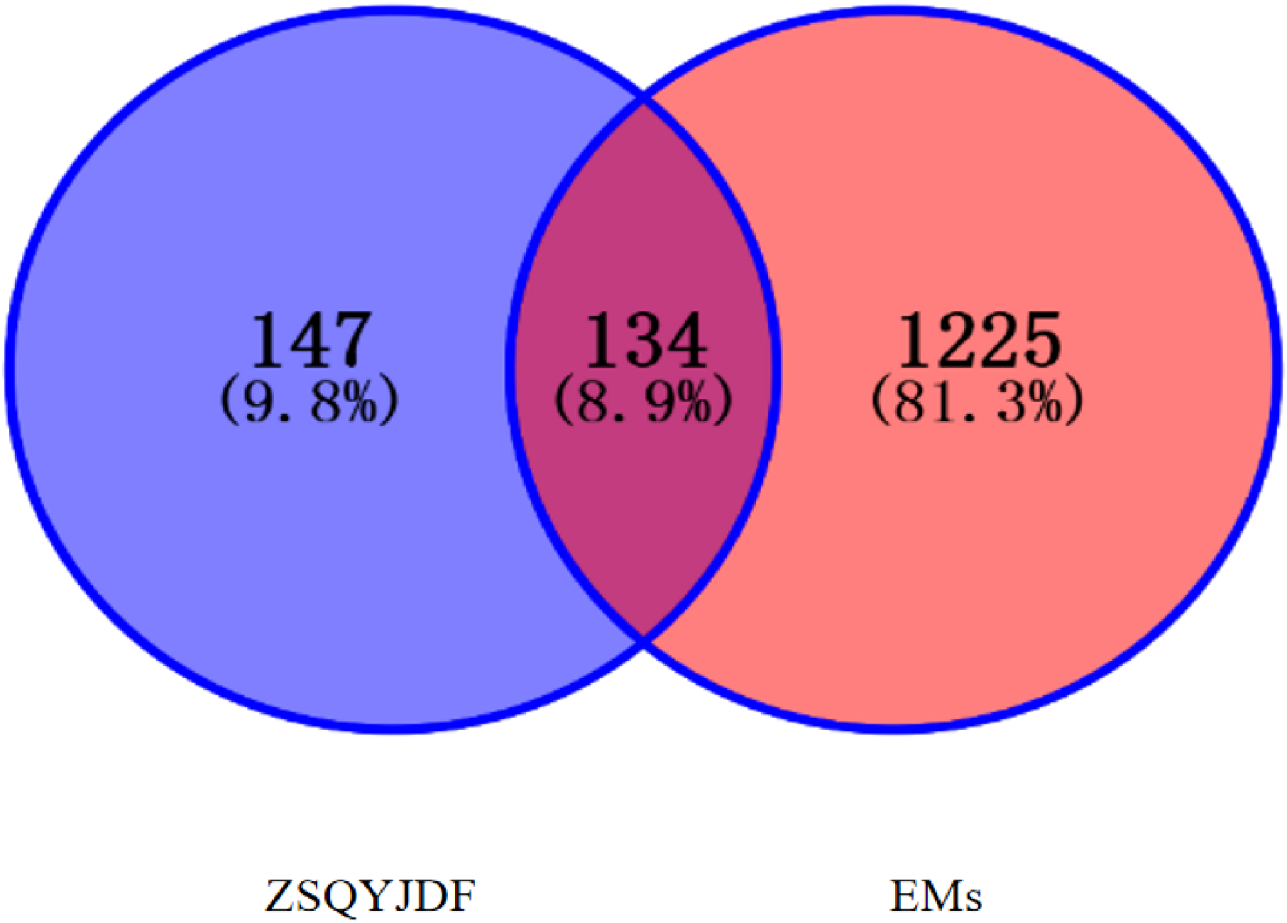
Venn diagram of ZSQYJDF and EMs targets.

From GeneCards, OMIM, and DRUGBANK databases, EMs-related targets were screened, yielding 1,255 targets in total.ZSQYJDF yielded 147 potential targets, of which 134 were associated with EMs. The distribution among constituent herbs included: Baishao 9, Chuanxiong 7, Daxueteng 5,Danshen65, Danggui 2, Sheng Dihuang 2, Gancao 92, Jinyinhua 23, Lianqiao 23, Meiguihua10, and others. After mapping the targets, the results identifying the potential targets of ZSQYJDF for treating the disease are shown in **Figure 3**.

#### 3.1.2 Construction of the ZSQYJDF–active compounds–target network

**Figure 4:**
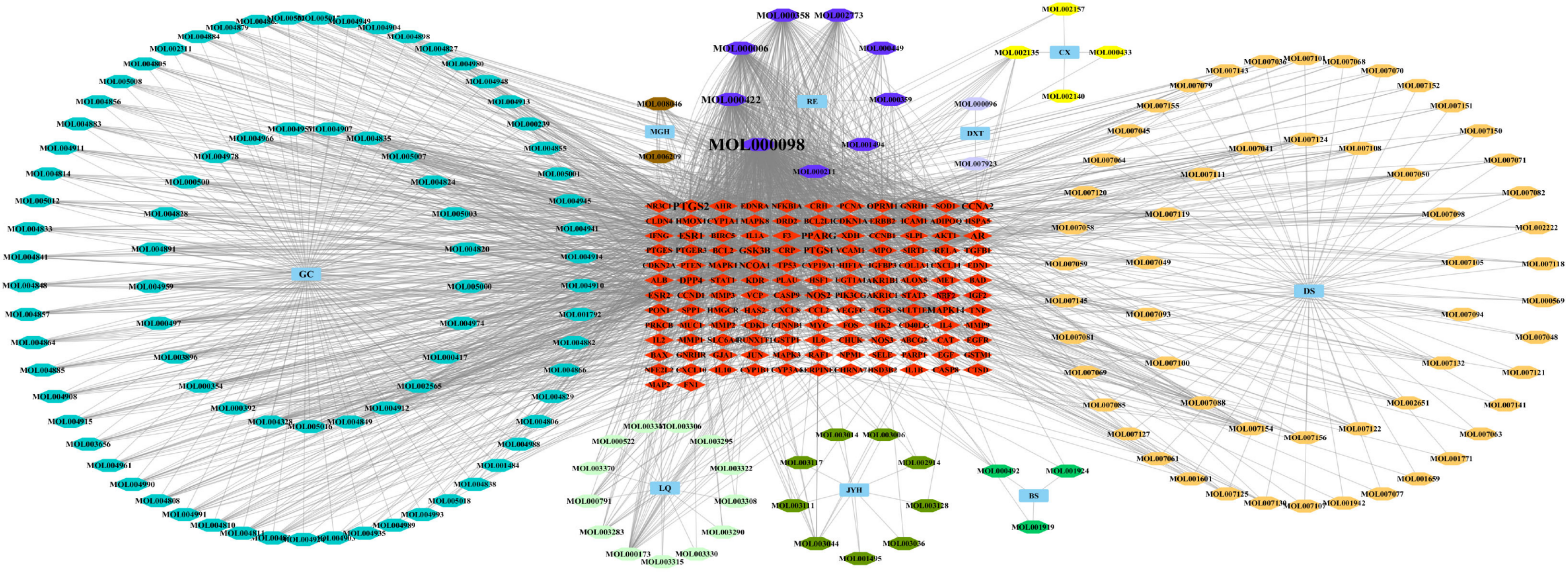
Network diagram of ZSQYJDF - Components - EMs Targets. Node Representations: GC, Zhigancao; MGH, Meiguihua; DXT, Daxueteng; CX, Chuangxiong; DS, Danshen; BS, Baishao; JYH, Jinyinhua; LQ, Lianqiao; RE, Shengdi、Danggui. Edge Representations: The lines (edges) connecting the nodes indicate the interactions between them.

After screening, a total of 1225 disease-related targets associated with EMs were identified. By intersecting the targets of EMs with those of ZQYJDF, 134 common targets were identified. These overlapping targets were imported into Cytoscape 3.7.1 to construct a drug-compound-target-disease network (**Figure 4**). In this network visualization, different node shapes and colors represent distinct types of elements: the ZQYJDF composition, active compounds, protein targets, and the disease (EMs). The connecting lines (edges) represent the interactions between these elements. The layout and visual attributes were configured to clearly illustrate the complex relationships between the formula composition and the disease.

#### 3.1.3 Construction of the PPI network

**Figure 5:**
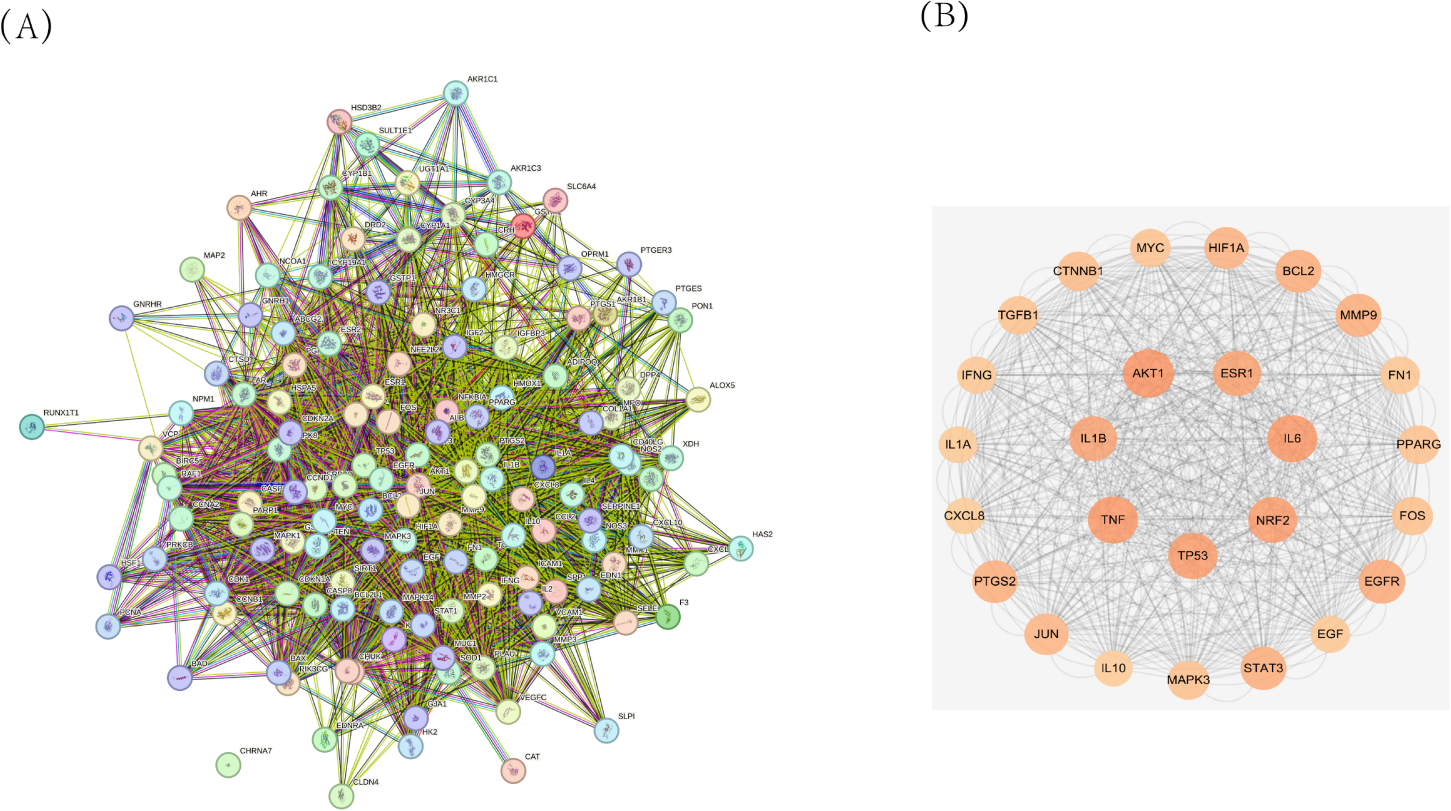
**(A)** Protein-Protein Interaction (PPI) network diagram of ZSQYJDF for treating EMs. **(B)** Core target protein diagram of ZSQYJDF for the treatment of EMs.

A protein–protein interaction (PPI) network was constructed using the screened targets (**Figure 5**). The network consisted of 134 nodes (28 targets were not displayed due to weak associations) and 7,328 interaction edges. The top seven core targets, AKT1, IL1B, TNF, TP53, NRF2, IL6, and ESR1, were identified (**Figure 5B**, **Table 6**). These results suggest that the formula exerts therapeutic effects by modulating these key targets.

**Table 6:** Summary of core targets.

No.	Target	Degree	Betweenness centrality
1	AKT1	110	0.027
2	ESR1	110	0.021
3	IL-6	108	0.027
4	NRF2	105	0.026
5	TP53	106	0.039
6	TNF	108	0.027
7	IL1B	102	0.022

#### 3.1.4 GO functional and KEGG pathway enrichment analysis

**Figure 6:**
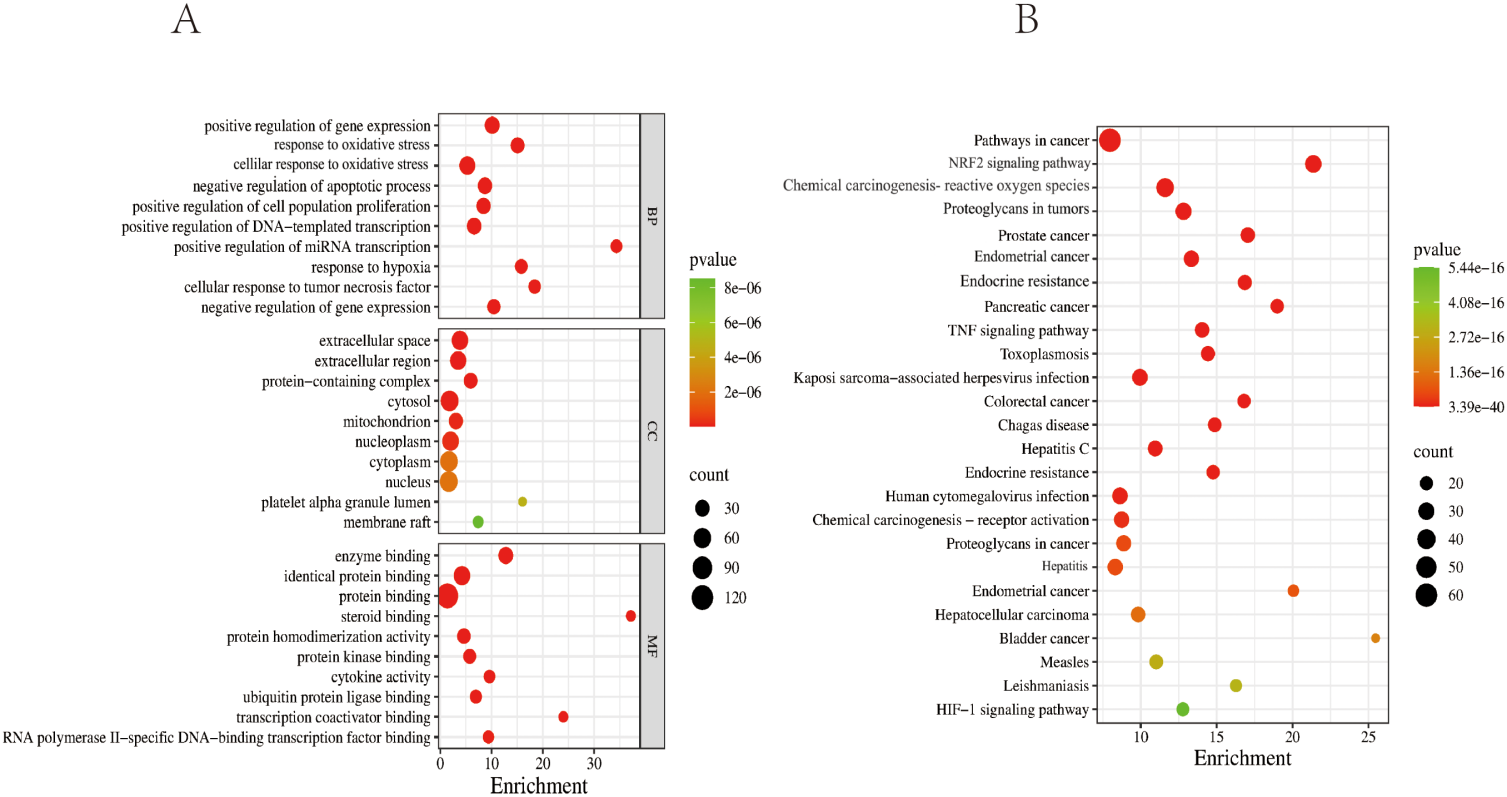
GO and KEGG enrichment analysis diagrams. **(A)** Top 10 GO terms for potential therapeutic targets of ZSQYJDF. The horizontal coordinate represents the gene ratio; the larger the point in the diagram, the more genes are involved. BP, biological process; CC, cellular component; MF, molecular function. **(B)** Top 20 KEGG pathways for potential therapeutic targets of the ZSQYJDF. The horizontal coordinate represents the gene ratio; the larger the point in the diagram, the more genes involved. P < 0.05 and FDR < 0.01.

The GO enrichment analysis results for biological processes (BP), cellular components (CC), and molecular functions (MF) were ranked by P-value. The significantly enriched BP terms mainly included positive regulation of gene expression, response to OS, cellular response to OS, positive regulation of transcription by RNA polymerase II, cell population proliferation, positive regulation of DNA-templated transcription, positive regulation of mRNA transcription, response to hypoxia, and cellular response to tumor necrosis factor. The enriched MF terms primarily involved enzyme binding, identical protein binding, steroid hormone receptor binding, protein homodimerization activity, protein kinase binding, and cytokine activity. The significantly enriched CC terms included the extracellular space, extracellular region, protein-containing complex, membrane raft, mitochondrion, nucleoplasm, cytoplasm, and platelet alpha granule lumen, as illustrated in **Figure 6A**.

The top 10 enriched KEGG pathways based on abundance were selected and visualized using a bubble plot (**Figure 6B**). The predominant pathways included pathways in cancer, the NRF2 signaling pathway, proteoglycans in cancer, the HIF-1 signaling pathway, and the TNF signaling pathway.

### 3.2 Compound analysis of ZSQYJDF

**Figure 7:**
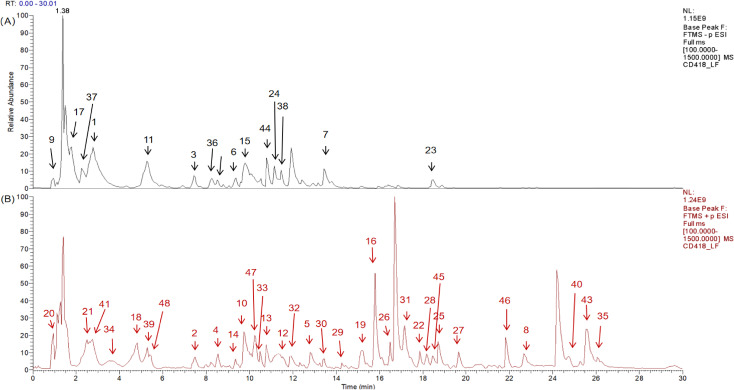
Qualitative analysis of ZSQYJDF sample by HPLC-Q-Orbitrap-MS, showing the total ion chromatograms in negative ion mode **(A)** and positive ion mode **(B)** for the ZSQYJDF sample.

In this study, a comprehensive analysis was conducted on the high-resolution mass spectrometry data obtained from the aqueous extract of ZSQYJDF in both the positive and negative ion modes. The analysis integrated multiple methods, including comparisons with reference standards, searches in a self-constructed database, and validation via the existing literature. A total of 48 compounds were identified or inferred, comprising 12 flavonoids, 10 phenolic acids and phenylpropanoids, 7 terpenoids, 4 organic acids and fatty acids, 8 nitrogen-containing compounds, 3 saccharides, and 2 others (such as anthraquinones and lignans). The detailed identification results are presented in **Table 7**. The total ion chromatograms and related analytical results of the aqueous decoction in both the positive and negative ion modes are shown in **Figure 7**.

**Table 7:** Identification of chemical constituents in ZSQYJDF by HPLC-Q-Orbitrap-MS.

No.	Formula	Ionmode	m/z (theoretical)	m/z (actual)	Weight	ppm	RT	Name	MS/MS	Classify	From	Reference
1	C_7_ H_6_ O_4_	[M-H]-	154.02547	153.01819	154.02661	-0.14	7.360	Protocatechuic acid	153.01828、135.00749、109.02809、108.98755	PhenolicAcidsand Phenylpropanoids	Danshen	(51)
2	C_16_ H_32_ O_2_	[M+H]+	273.26656	274.27383	276.24023	2.2	15.193	Palmitic Acid	255.23279、256.23761、120.90842、91.96030	PhenolicAcidsand Phenylpropanoids	Danshen	(51)
3	C_18_ H_36_ O_2_	[M-H]-	284.27167	283.2644	284.27153	0.14	23.928	Stearic acid	283.26440、244.65749、203.07108、146.98137	OrganicAcids and Fatty Acids	Danshen	(51)
4	C_16_ H_32_ O_2_	[M+H]+	273.26656	274.27383	256.24023	-0.18	15.193	Palmitic Acid	255.23279、256.23279、120.90842	OrganicAcids and Fatty Acids	Danshen	(51)
5	C_19_ H_18_ O_3_	[M+H]+	294.12551	295.13281	294.12559	-0.8	18.097	Tanshinone IIA	296.13281、280.10950、277.12238、249.12727、235.07526、221.13251、207.08058、206.10901	Terpenoids	Danshen	(51)
6	C_7_ H_6_ O_3_	[M-H]-	138.03151	139.03883	138.03169	-0.8	11.363	3,4-Dihydroxybenzaldehyde	139.03894、111.04437、95.0867、83.04979、79.05488	PhenolicAcids and Phenylpropanoids	Danshen	(51)
7	C_5_ H_5_ N_5_	[M-H]-	135.05333	134.04605	135.0545	1.5	9.788	Adenine	136.06178、119.03548、112.05118、92.02491	Nitrogen-Containing Compounds	Danshen	(51)
8	C_4_H_4_ N_2_ O_2_	[M+H]+	112.02771	113.03499	112.02728	0.3	17.68	Uracil	113.03487、113.02386、96.00853、95.04964、	Nitrogen-Containing Compounds	Danshen	(51)
9	C_8_ H_8_ O_4_	[M-H]-	168.04136	167.03407	168.04226	-0.9	6.678	Vanillic acid	169.04948、151.03902、125.05980、123.04420、111.04435、95.04964、93.03400	PhenolicAcids and Phenylpropanoids	Danshen	(51)
10	C_19_ H_20_ O_3_	[M+H]+	296.14095	297.14822	296.14124	-0.3	18.727	Cryptotanshinone	297.14847、282.12503、254.09366、251.14287	Terpenoids	Danshen	(51)
12	C_15_ H10 O5	[M+H]+	270.05266	271.05978	270.05282	1.7	15.729	Cryptotanshinone	297.14847、279.13809、268.10934、254.09366、251.14287	Anthraquinones	Daxueteng	(52)
13	C15 H10 O7	[M+H]+	302.04238	303.04965	302.04265	1.1	12.919	Quercetin	303.04974、304.05301、228.04169、155.04890、137.02341	Flavonoids	Daxueteng	(52)
14	C15 H10 O6	[M+H]+	286.04782	287.05496	286.04774	-0.27	15.094	Kaempferol	287.05490、269.09189、223.08650、195.09192、180.08072、153.01828	Flavonoids	Daxueteng	(52)
15	C6 H8 O7	[M-H]-	192.02635	191.01907	192.02701	-0.74	2.779	citric acid	191.0188、129.01799、111.00735、87.00726、85.02799	OrganicAcids and Fatty Acids	Jinyinhua	(53)
16	C21 H20 O11	[M+H]+	448.10038	449.10754	449.10454	-0.51	12.721	Cynaroside	449.10834、296.04398、161.02342、153.01817、135.04408、68.99786	Flavonoids	Jinyinhua	(53)
17	C16 H18 O9	[M-H]-	354.09444	355.10168	354.09508	-0.6	11.757	Cryptochlorogenic acid	163.03891、135.04411、117.03381、79.05494、83.0498、63.0241	PhenolicAcids and Phenylpropanoids	Jinyinhua	(53)
18	C15 H10 O5	[M+H]+	270.05266	271.05978	270.05282	-0.7	15.729	Apigenin	271.09677、229.08582、161.03932、121.02869	Flavonoids	Jinyinhua	(53)
19	C27 H30 O14	[M+H]+	578.1633	579.17047	578.16356	-0.25	13.327	Rhoifolin	579.21857、579.17047、580.17413、368.11243、271.05984	Flavonoids	Jinyinhua	(53)
20	C30 H48 O3	[M+H]+	456.36027	457.36742	456.36035	-0.08	22.112	Ursolic acid	501.35876 455.35309、456.36651、 407.33115、	Terpenoids	Jinyinhua	(53)
21	C35H56O7	[M+H]+	456.36035	456.36047	456.38066	1.8	21.877	Oleanolic acid	439.35715、393.35114、203.17949、191.17946	Terpenoids	Jinyinhua	(53)
22	C10 H10 O4	[M+H]+	194.05792	195.06525	194.05791	-0.7	13.173	Methyl caffeate	195.06520、163.03889、145.02847、135.04411、117.03380	PhenolicAcids and Phenylpropanoids	Jinyinhua	(53)
23	C16 H18 O9	[M-H]-	354.09529	353.08801	354.09508	-0.6	9.525	Neochlorogenic acid	353.08801、192.05908、191.05527、179.03406、173.04463、135.04388	PhenolicAcids and Phenylpropanoids	Jinyinhua	(53)
24	C10 H16 O	[M-H]-	152.12009	153.12733	152.12012	0.3	12.08	Citral	153.12732、135.11685、107.08590、93.07042、95.08604	Terpenoids	Jinyinhua	(53)
25	C15 H10 O6	[M+H]+	286.04782	287.05496	286.04774	0.08	15.094	Luteolin	287.05496、241.09738、223.08650、180.08072	Flavonoids	Jinyinhua	(53)
26	C27H30 O16	[M+H]+	610.15287	610.15287	610.15338	-0.5	12.915	Rutin	465.10248、303.04971、247.06020、85.02906、71.04994	Flavonoids	Jinyinhua	(53)
27	C27H34 O11	[M+H]+	551.23613	552.24341	534.21011	1.7	13.43	Arctiin	552.24341、337.14294、219.10117、201.09087	Lignans	Jinyinhua	(53)
28	C9 H8 O4	[M+H]+	162.0315	163.03877	162.03169	-0.2	10.228	Caffeic acid	179.03406、135.04388、133.02838	PhenolicAcids and Phenylpropanoids	Jinyinhua	(53)
29	C5 H11 N O2	[M+H]+	118.08641	117.07908	117.07898	0.1	1.374	Betaine	118.08641、91.97030、58.06606、59.07389	Nitrogen-Containing Compounds	Gouqizi	(54)
30	C12H22 O11	[M-H]-	342.11568	387.11389	342.11621	-0.54	10.488	Sucrose	864.64307、443.22919、327.14294、267.12265、197.08093	Carbohydrates	Gouqizi	(54)
31	C18 H34 O2	[M-H]-	282.25593	281.24866	282.25588	0.5	22.861	Oleic Acid	281.24875、282.25186	OrganicAcids and Fatty Acids	Gouqizi	(54)
32	C6 H5 N O2	[M+H]+	123.03237	124.03964	123.03203	0.34	8.148	Nicotinic acid	124.03947、106.02950、96.04494、80.05017、78.03454、53.03954	Nitrogen-Containing Compounds	Gouqizi	(54)
33	C12 H16 O4	[M+H-]+	224.10472	223.10144	224.10486	-0.14	13.235	Senkyunolide H	207.10141、189.09096、165.05461、133.10115、119.08575	PhenolicAcids and Phenylpropanoids	Chuanxiong	(54)
34	C10 H10 O4	[M+H]+	194.05724	193.04991	194.05791	-0.067	11.843	Ferulic acid	193.04991、178.02617、134.03606	PhenolicAcids and Phenylpropanoids	Chuanxiong	(54)
35	C10 H10 O2	[M+H]+	162.04188	163.07532	163.08542	-32.026	8.499	Safrol	180.10185、145.02847、131.04926、117.07008、103.05470	PhenolicAcids and Phenylpropanoids	Lianqiao	(55)
36	C6 H13 O9 P	[M-H]-	260.0295	259.02225	260.02972	-0.022	7.738	D-Glucose 6-phosphate	259.01389、241.01140、138.96948、96.96825、78.95754	Carbohydrates	Shengdihuang	(56)
37	C18H32 O16	[M-H]-	504.16866	549.16687	504.16903	-0.027	11.362	D-Raffinose	533.20276、325.111267、163.06006、145.04953、127.03912、85.02907	Carbohydrates	Tusizi	(57)
38	C6H9 N3 O2	[M-H]-	455.06832	454.06094	155.06948	-0.016	12.271	Histidine	154.06108、137.03441、136.05022、110.07103、93.00436	Nitrogen-Containing Compounds	Tusizi	(57)
39	C6H14N4O2	[M+H]+	174.11162	175.1189	174.11168	-0.8	10.255	Arginine	175.11899、158.09244、116.07091、70.06594、60.05653	Nitrogen-Containing Compounds	Tusizi	(57)
40	C3 H7 N O3	[M+H]+	105.04259	105.04105	104.03378	0.28	1.282	Serine	105.03034、96.67028、92.02036、64.10708	Nitrogen-Containing Compounds	Tusizi	(57)
41	C9H11 N O3	[M+H]+	381.07421	382.08148	382.08557	0.29	12.103	Tyrosine	182.08148、165.05467、136.07576、123.04429、96.04496、87.04472	Nitrogen-Containing Compounds	Tusizi	(57)
42	C21H20 O12	[M+H]+	464.09536	465.10245	464.09548	-0.11	12.919	Hyperoside	487.08441、465.10245、303.04956	Flavonoids	Tusizi	(57)
43	C16 H12 O4	[M+H]+	268.07338	269.0806	268.07356	1.8	15.877	Formononetin	269.08060、254.05708、213.09082	Flavonoids	Zhigancao	(58)
44	C15 H12 O5	[M-H]-	272.06871	271.06143	272.06847	0.04	14.525	Naringenin	271.06137、177.01837、151.00249、119.04887、107.01241	Flavonoids	Zhigancao	(58)
45	C15 H12 O4	[M+H]+	256.07323	257.08051	256.07356	-0.33	11.878	Isoliquiritigenin	257.08063、222.07529、163.03891、119.04942、91.05482、81.03423	Flavonoids	Zhigancao	(58)
46	C30 H46 O4	[M+H]+	470.33949	471.34677	470.33961	1.2	18.874	18-β-Glycyrrhetinic acid	471.34732、425.34177、217.15910、175.14818	Terpenoids	Zhigancao	(58)
47	C16 H12 O7	[M+H]+	316.05806	317.06534	316.0583	-0.24	13.676	Isorhamnetin	317.06537、274.04684、228.04152、165.01831、139.03883	Flavonoids	Zhigancao	(58)
48	C23H28 O11	[M+H]+	480.16262	481.16986	480.16316	-0.54	10.064	Albiflorin	481.17007、301.10684、197.08076、161.05960、105.03387	Terpenoids	Baishao	(59)

### 3.3 Animal experimental results

#### 3.3.1 Effect of ZSQYJDF on the size of ectopic lesions in rats

The body weights of the rats in each group were recorded after modeling. No statistically significant differences in body weight were observed among the groups at the time points of 1, 7, 14, 21days (P > 0.05). Evaluation of the longest diameter of ectopic lesions showed that both the mifepristone and ZSQYJDF groups exhibited a significant reduction in the volume of ectopic endometrial tissue compared to the model group. (**Table 8**).

**Table 8:** Comparison of ectopic endometrium volume in rats of each group.

Groups	Volume of lesions(mm3)
Blank Control	–
Model	110.49 ± 18.42
ZSQYJDF	44.68 ± 12.27
Mifepristone	40.81 ± 14.46

#### 3.3.2 Effect of ZSQYJDF on histopathology of ectopic tissue in rats

**Figure 8:**
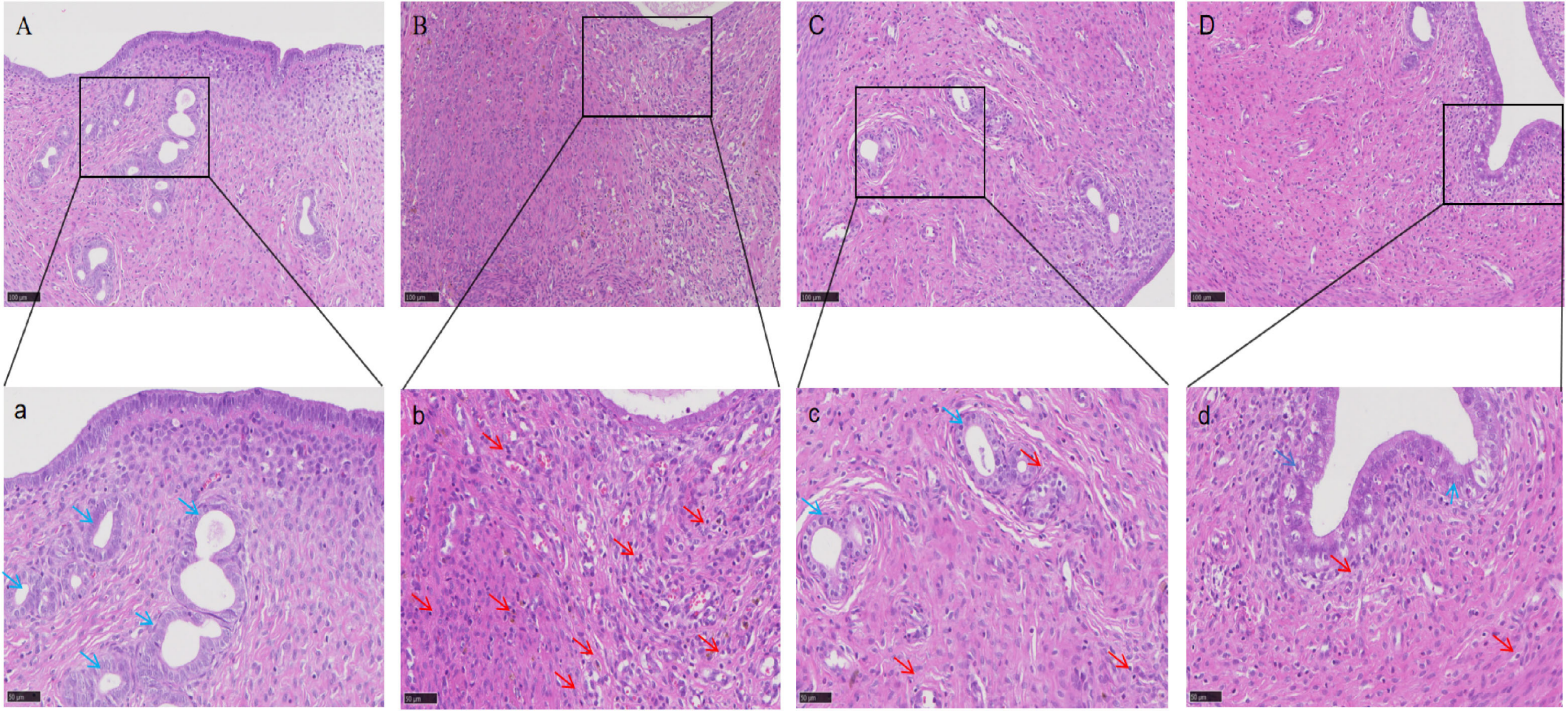
HE staining results of uterine tissue from rats in each group (100×, 200×). n=6. **(A)** Blank group; **(B)** Model group; **(C)** ZSQYJDF group; **(D)** Mifepristone group (100×). **(a)** Blank group **(b)** Model group **(c)** ZSQYJDF group **(d)** Mifepristone group (200×). Red arrows: inflammatory cell infiltration; Blue arrows: glands.

Histopathological examination of rat endometrial tissue revealed that in the blank group, under light microscopy, a significant amount of endometrial and uterine glandular epithelium was observed, with thickened lamina propria, increased stromal cells, and regularly arranged glands without pathological changes. In the model group, the ectopic endometrium of rats with EMs showed increased thickness, reduced glands, and extensive cellular infiltration. Compared with the blank group, the model group exhibited significant pathological alterations in tissue morphology, cellular composition and inflammatory response. These characteristics indicate the successful modeling of EMs. In both the ZSQYJDF and mifepristone groups, the thickness of the ectopic endometrium was notably reduced, the number of glandular epithelium was comparable to that of the model group, and cellular infiltration was decreased. These results demonstrate that ZSQYJDF can reduce local inflammatory cell infiltration in ectopic lesions, thereby alleviating pathological endometrial thickening and tissue disorder, and ultimately achieving therapeutic effects against EMs (**Figure 8**).

#### 3.3.3 Effect of ZSQYJDF on serum inflammatory cytokine levels

**Figure 9:**
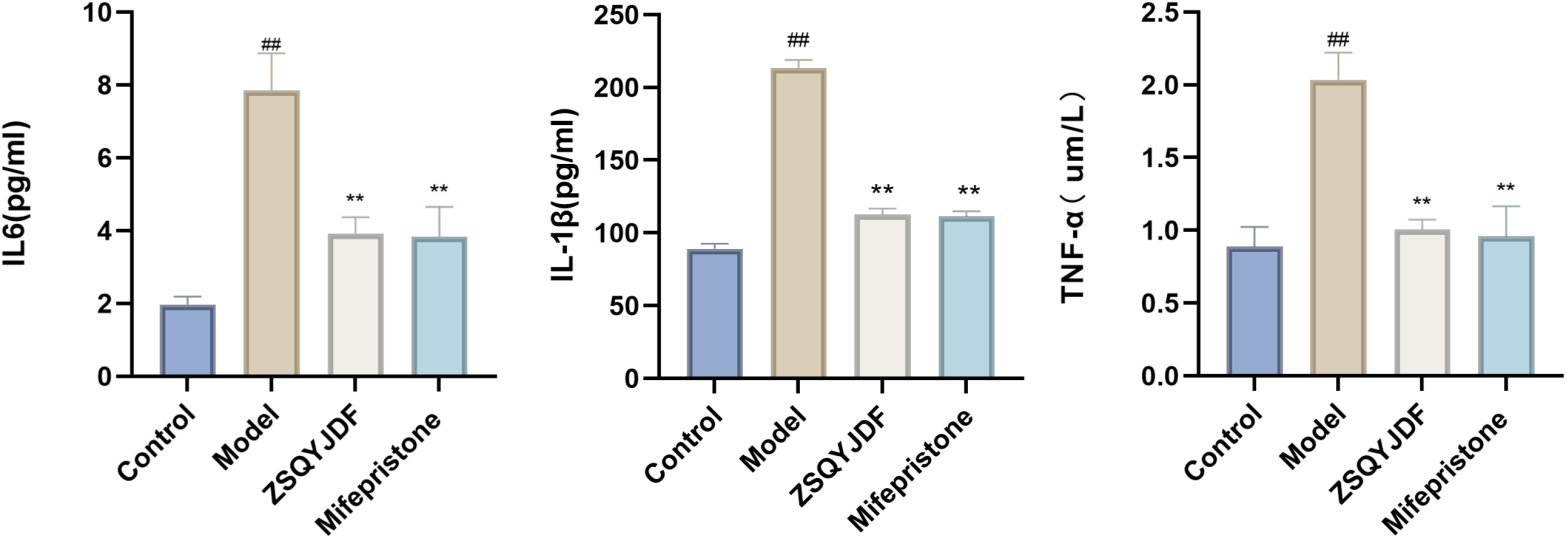
Histograms of TNF-α, IL-1β, and IL-6 levels in the serum of rats from each group. n=6; all data are expressed as mean ± standard deviation. Statistical significance was determined using one-way ANOVA combined with Tukey’s *post hoc* test. # compared with the blank group, * compared with the model group; ##P<0.01; **P < 0.01. For TNF-α, the P-value for the blank group vs. the model group was P=0.004; the P-values for the model group vs. the ZSQYJDF group and mifepristone group were P=0.007 and P=0.006, respectively. For IL-1β, the P value for the blank group vs. model group was P=0.001; the P values for the model group vs. ZSQYJDF group and mifepristone group were P=0.004 and P=0.038, respectively. For IL-6, the P value for the blank group vs. model group was P=0.005; the P values for the model group vs. ZSQYJDF group and mifepristone group were P=0.045 and P=0.005, respectively.

To evaluate the ameliorative effect of ZSQYJDF on the inflammatory response in the EMs rat model, the expression levels of inflammatory cytokines in the serum were measured using ELISA. Compared to the control group, the model group showed significantly elevated serum levels of TNF-α, IL-1β, and IL-6 (P < 0.01). Compared to the model group, the ZSQYJDF group exhibited significantly reduced levels of TNF-α, IL-1β, and IL-6 (P < 0.05). Similarly, the mifepristone group also demonstrated significantly decreased levels of these pro-inflammatory cytokines relative to the model group (P < 0.05). These results suggest that ZSQYJDF can reduce the transcription and release of pro-inflammatory cytokines, such as TNF-α, IL-1β, and IL-6, potentially by modulating the NRF2-mediated antioxidant–anti-inflammatory network, thereby significantly alleviating systemic inflammation and contributing to its therapeutic effects on EMs (**Figure 9**).

#### 3.3.4 Effect of ZSQYJDF on serum oxidative stress markers

**Figure 10:**
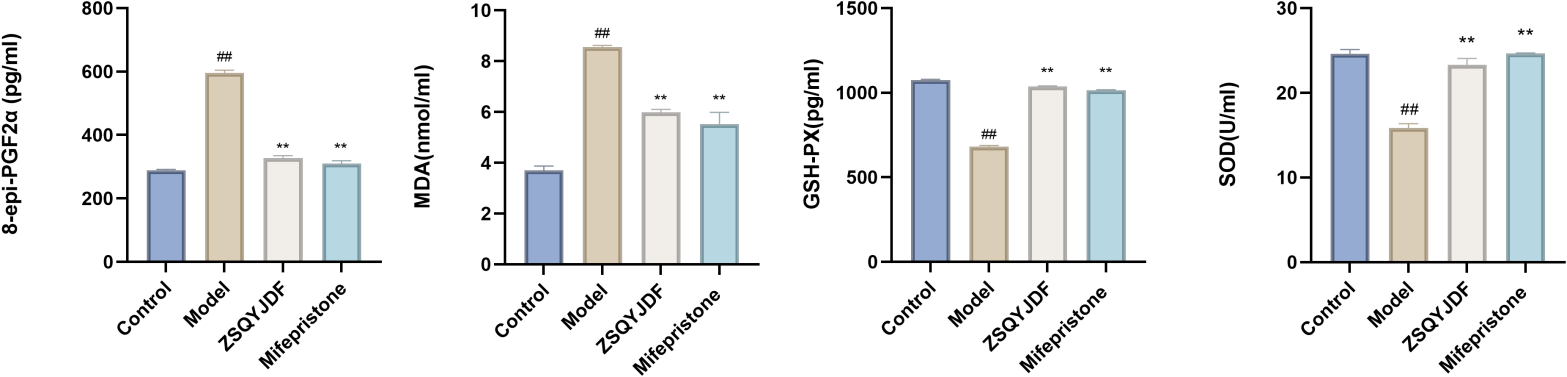
Expression levels of 8-epi-PGF2α, MDA, SOD, and GSH-PX in the serum of rats from each group. n=6; all data are expressed as mean ± standard deviation. Statistical significance was determined using one-way ANOVA combined with Tukey’s *post hoc* test. # compared with the blank group, compared with the model group; ##P<0.01; **P < 0.01. For 8-epi-PGF2α, the P value for the blank group vs. the model group was P=0.001; the Exact P values for the model group vs. the ZSQYJDF group and mifepristone group were P=0.004 and P=0.004, respectively. For MDA, the P value for the blank group vs. the model group was P=0.001; the Exact P values for the model group vs. the ZSQYJDF group and mifepristone group were P=0.004 and P=0.038, respectively. For SOD, the P value for the blank group vs. the model group was P=0.003; the Exact P values for the model group vs. the ZSQYJDF and mifepristone group were P=0.005 and P=0.004, respectively. For GSH-PX, the P value for the blank group vs. model group was P=0.015, the and P values for the model group vs. ZSQYJDF group and mifepristone group were P=0.009 and P=0.007, respectively.

To investigate the regulatory effect of ZSQYJDF on OS during EMs progression, we measured the serum levels of oxidative damage markers (MDA and 8-epi-PGF2α) and antioxidant enzymes (SOD and GSH-PX) in each group. ELISA results showed that the model group had significantly increased levels of MDA and 8-epi-PGF2α compared to the control group (P < 0.01), indicating enhanced lipid peroxidation and OS. Meanwhile, the activities of SOD and GSH-PX in the model group were significantly lower than those in the control group (P < 0.01), suggesting impaired endogenous antioxidant defense and confirming a significant redox imbalance in EMs model rats. Compared with the model group, both the mifepristone and ZSQYJDF groups showed significantly reduced levels of MDA and 8-epi-PGF2α (P < 0.05), as well as significantly increased activities of SOD and GSH-PX (P < 0.05). These results indicate that the EMs model successfully induced significant OS and lipid peroxidation damage.ZSQYJDF may alleviate OS in EMs by modulating the NRF2-mediated antioxidant defense system, enhancing the ability to scavenge ROS, inhibiting lipid peroxidation, and restoring homeostasis of the oxidative–antioxidative system, thereby delaying disease progression. (**Figure 10**).

#### 3.3.5 Effect of ZSQYJDF on the expression and localization of NRF2, HO-1, and NQO1 proteins in ectopic endometrium as determined by immunohistochemistry

**Figure 11:**
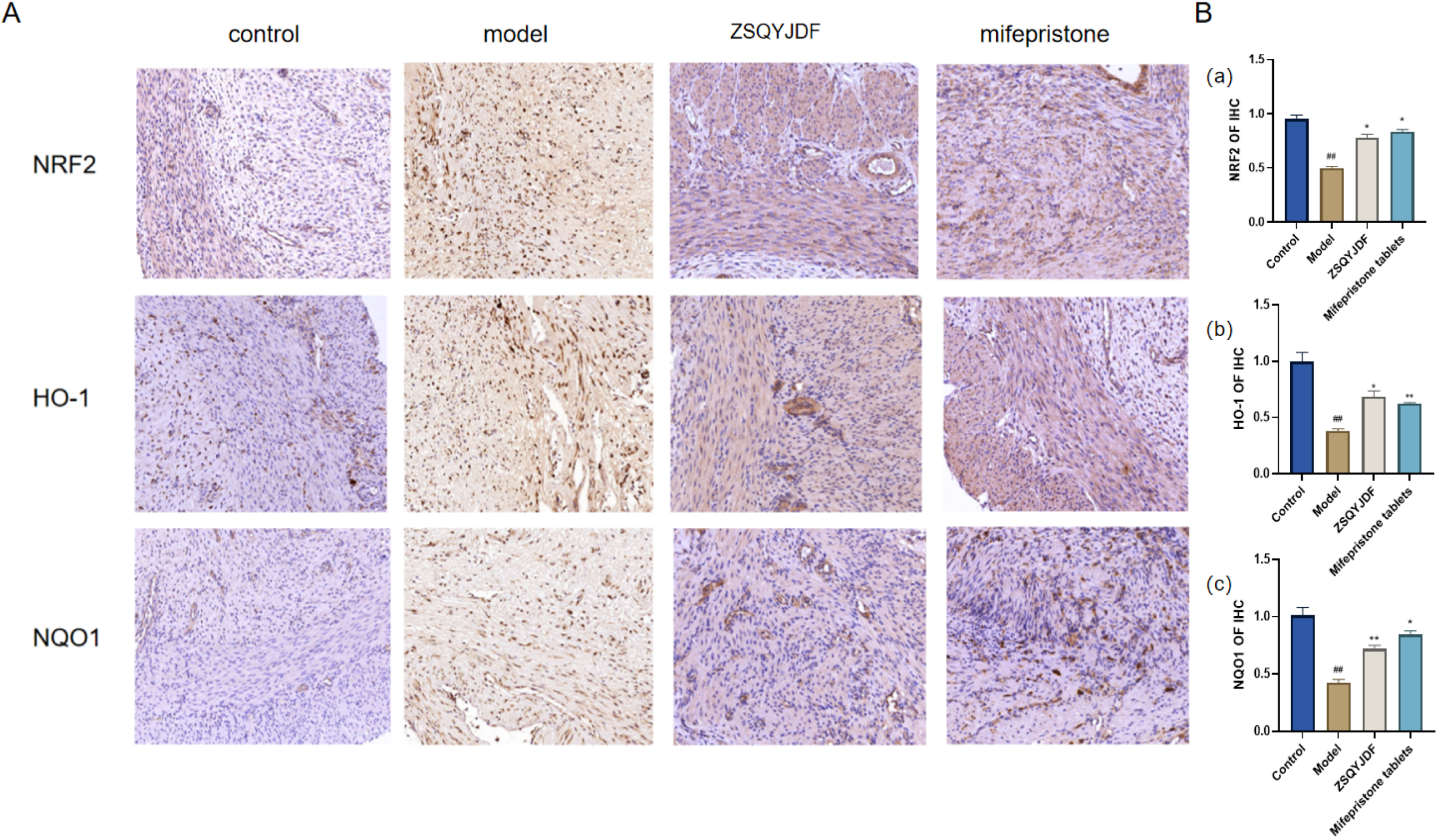
**(A)** Comparison of positive protein expression of NRF2, NQO1, and HO-1 in the ectopic endometrium of rats from each group (immunohistochemical staining, 200×). **(B)** Histogram of positive protein expression of NRF2, NQO1, and HO-1 in the ectopic endometrium of rats from each group (a) NRF2 (b) NQO1 (c) HO-1. n = 6; all data are expressed as mean ± standard deviation. Statistical significance was determined using one-way ANOVA combined with Tukey’s *post hoc* test. # compared with the blank group, * compared with the model group; #P<0.05, ##P<0.01; *P<0.05, P<0.01. For NRF2, P value for blank group vs. model group was P=0.001; P values for model group vs. ZSQYJDF group and mifepristone group were: P=0.011, P=0.019, respectively. For HO-1, P value for blank group vs. model group was: P=0.007; P values for model group vs. ZSQYJDF group and mifepristone group were: P=0.0085, P=0.0298, respectively. For NQO1, P value for blank group vs. model group was: P=0.005; P values for model group vs. ZSQYJDF group and mifepristone group were: P=0.0058, P=0.0065, respectively. **: P<0.01.

Immunohistochemistry was performed to detect the expression of NRF2, HO-1, and NQO1. Positive staining for NRF2, HO-1, and NQO1 was observed as brownish-yellow signals. NRF2 is primarily localized in the nucleus and cytoplasm, whereas HO-1 and NQO1 (30)are mainly expressed on the cell membrane. Compared with the control group, the model group showed significantly lower positive expression rates of NRF2, HO-1, and NQO1 in ectopic endometrial tissue (P < 0.01), indicating impaired antioxidant defense and insufficient activation of the NRF2 signaling pathway under pathological conditions of EMs, which restricts the transcription of downstream antioxidant genes. In contrast, both the ZSQYJDF and mifepristone groups exhibited significantly increased positive expression of NRF2, HO-1, and NQO1 compared with the model group (P < 0.05). The nuclear accumulation of NRF2 protein suggests effective activation of this pathway, leading to the transcription of cytoprotective genes such as HO-1 and NQO1. These results suggest that ZSQYJDF may alleviate oxidative damage in ectopic endometrial tissue and exert therapeutic effects on EMs by activating the NRF2 signaling pathway and enhancing antioxidant capacity (**Figure 11**).

#### 3.3.6 Effect of ZSQYJDF on the protein expression levels of NRF2, HO-1, NQO1, and KEAP1 in ectopic endometrium

**Figure 12:**
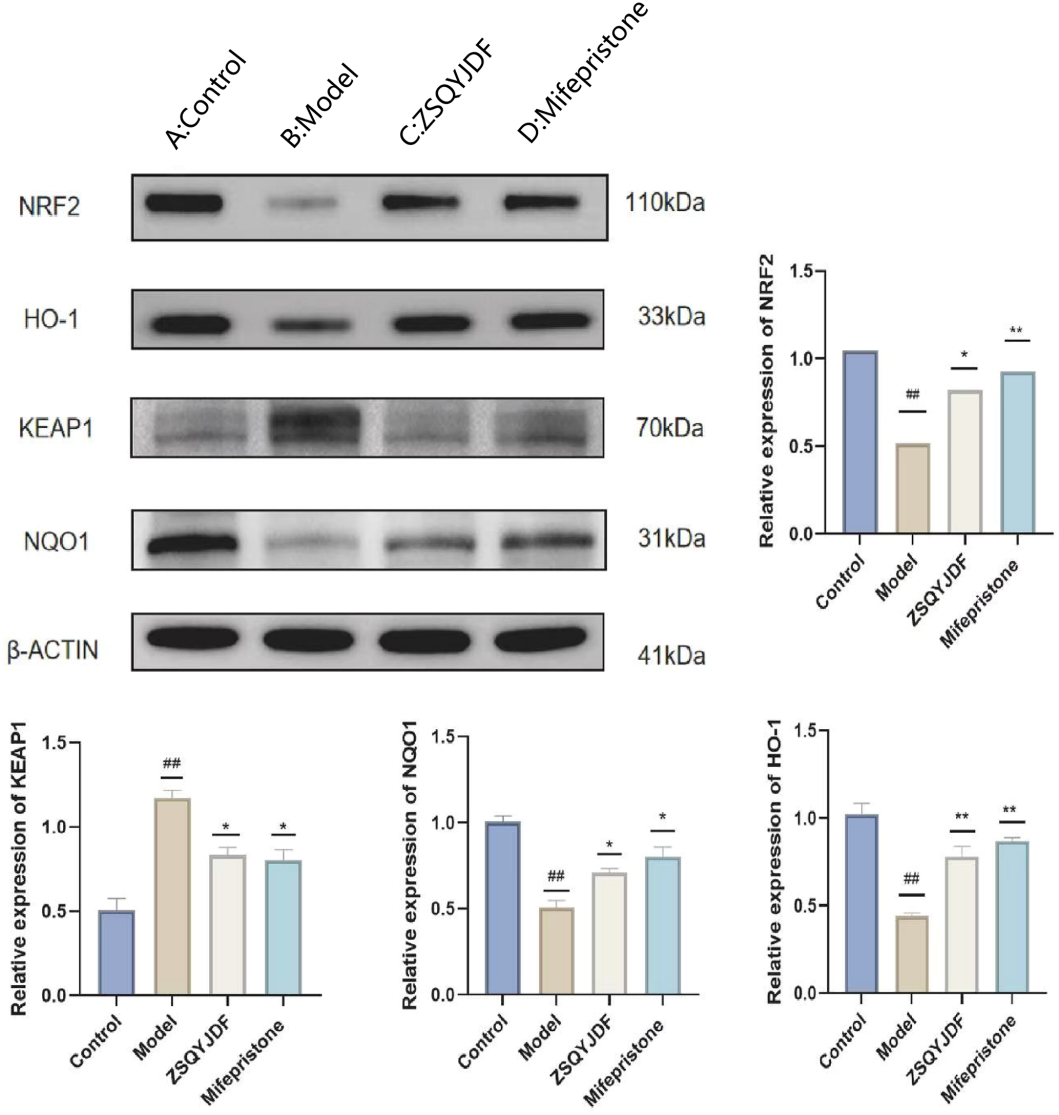
Effect of ZSQYJDF on the expression of proteins related to the NRF2/HO-1 signaling pathway. **(A)** NRF2, HO1, NQO1, and KEAP1. **(a)** Protein expression of NRF2 **(b)** Protein expression of KEAP1 **(c)** Protein expression of HO-1 **(d)** Protein expression of NQO1. n=3; all data are expressed as mean ± standard deviation; statistical significance was determined using one-way ANOVA combined with Tukey’s *post hoc* test. # compared with the blank group, * compared with the model group; #P<0.05, ##P<0.01; *P<0.05, P<0.01; Comparing model group with blank group, NRF2 (P=0.004), HO1 (P=0.001), NQO1 (P=0.001), KEAP1 (P=0.002); Comparing mifepristone group and ZSQYJDF group with model group, NRF2 (P=0.012, P=0.002), HO1 (P=0.022, P=0.015), NQO1 (P=0.034, P=0.041), KEAP1 (P=0.013, P=0.021). **: P<0.01.

To investigate whether ZSQYJDF exerts its therapeutic effect by regulating the antioxidant signaling pathway, we measured the protein expression levels of NRF2, HO-1, NQO1, and KEAP1 in endometrial tissues from each group using western blotting. NRF2 is one of the most critical antioxidant factors in the human body. It interacts with its inhibitory protein, KEAP1, to activate the transcription of various cytoprotective genes, thereby maintaining redox homeostasis (31). The results showed that compared with the control group, the model group exhibited significantly downregulated protein expression of NRF2, HO-1, and NQO1 (P < 0.01), while KEAP1 expression was increased (P < 0.01). In contrast, both the mifepristone and ZSQYJDF groups showed significantly increased expression of NRF2, HO-1, and NQO1 (P < 0.05) and decreased expression of KEAP1 (P < 0.05) compared with the model group. Downregulation of KEAP1 relieves its inhibitory effect on NRF2, promoting NRF2 nuclear translocation and transcriptional activity, and subsequently upregulating the expression of antioxidant proteins, such as HO-1 and NQO1, thereby enhancing cellular resistance to OS. These results suggest that ZSQYJDF may enhance the endogenous antioxidant defense capacity by inhibiting KEAP1 expression and activating the NRF2–HO-1 signaling axis, thereby alleviating oxidative damage in ectopic endometrial tissue. This mechanism could be one of the key ways in which the formula treats EMs (**Figure 12**).

#### 3.3.7 Effect of ZSQYJDF on the mRNA expression levels of NRF2, HO-1, NQO1, and KEAP1

**Figure 13:**
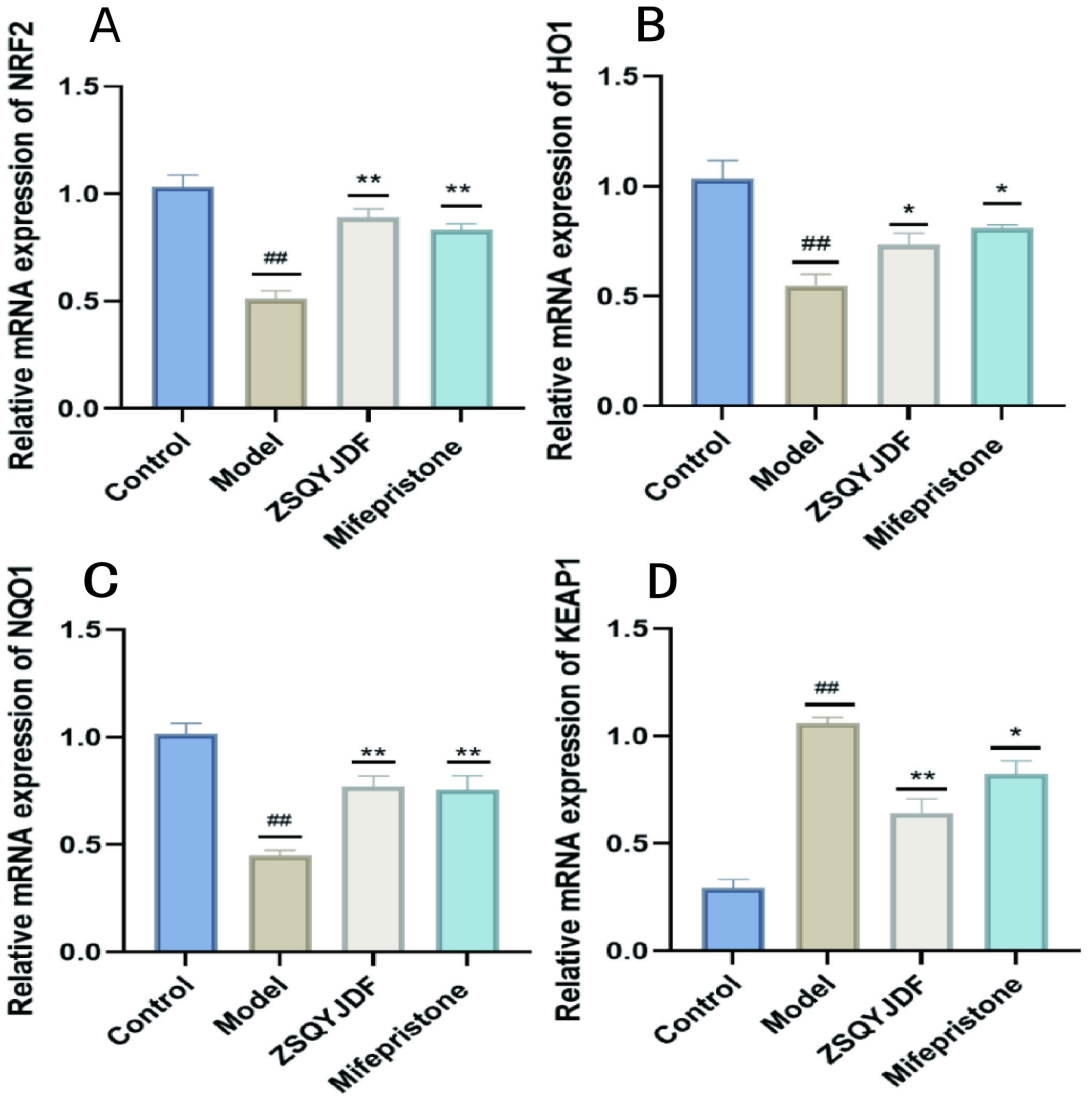
Comparison of mRNA expression of NRF2, NQO1, HO-1, and KEAP1 in the endometrium of rats from each group treated with ZSQYJDF. **(A)** mRNA expression of NRF2, **(B)** mRNA expression of HO-1, **(C)** mRNA expression of NQO1, and **(D)** mRNA expression of KEAP1. n = 3; all data are expressed as mean ± standard deviation. Statistical significance was determined using one-way ANOVA combined with Tukey’s *post hoc* test. # compared with the blank group, * compared with the model group; ##P<0.01; *P<0.05, **p < 0.01. Compared with the blank group, the model group showed NRF2 (P=0.001), HO1 (P=0.003), NQO1 (P=0.001), KEAP1 (P=0.001); Compared with the model group, the ZSQYJDF group and mifepristone group showed NRF2 (P=0.049, P=0.027), HO1 (P=0.049, P=0.045), NQO1 (P=0.0069, P=0.0091), KEAP1 (P=0.017, P=0.028). **: P<0.01.

The NRF2/HO-1 signaling pathway is a core regulatory pathway through which cells respond to OS. To clarify the intervention effect of ZSQYJDF on this pathway at the transcriptional level, we used q-PCR technology to detect the mRNA expression levels of NRF2, HO-1, NQO1, and KEAP1 in ectopic endometrial tissues of rats from each group. PCR results showed that compared with the blank control group, the mRNA expressions of NRF2, HO-1, and NQO1 were significantly decreased in the model group (P < 0.01), while KEAP1 mRNA was increased (P < 0.05), indicating that under EMs conditions, the NRF2 signaling pathway is inhibited at the gene transcription level and the expression of antioxidant genes is impaired. Compared with the model group, the mRNA expressions of NRF2, HO-1, and NQO1 were significantly increased in the ZSQYJDF and mifepristone groups (P < 0.01), whereas KEAP1 expression was downregulated (P < 0.05). As a negative regulator of NRF2, decreased expression of KEAP1 alleviates its ubiquitin-mediated degradation of NRF2, promotes NRF2 protein stabilization and nuclear translocation, and thereby initiates the transcription of downstream cytoprotective genes such as HO-1 and NQO1. These results indicate at the mRNA level that ZSQYJDF can comprehensively activate the NRF2/HO-1 antioxidant signaling pathway by inhibiting KEAP1 transcription and promoting the expression of NRF2 and its target genes, HO-1 and NQO1, thereby enhancing the body’s endogenous antioxidant capacity and effectively alleviating the OS state in EMs. (**Figure 13**).

## 4 Discussion

In recent years, the incidence of EMs has been on a continuous rise. According to the latest data and reports from the Chinese Gynecological Disease Diagnosis and Treatment Center, approximately 10%-15% of women of childbearing age worldwide suffer from this disease, corresponding to about 190 million patients; in China, the number of patients has exceeded 60 million, with a significant increase in the incidence among young people (32, 33). Our previous clinical studies have confirmed that ZSQYJDF can effectively improve the clinical symptoms and inflammatory response of EMs patients, indicating its good therapeutic potential (24–27).This study further revealed through *in vivo* experiments that ZSQYJDF can significantly inhibit the growth of ectopic lesions in EMs model rats, and its mechanism is closely related to activating the NRF2/HO-1 signaling pathway and enhancing antioxidant defense capacity. With the activation of this pathway, the levels of OS markers and inflammatory factors in rats were significantly reduced, and the pathological state of ectopic lesions was significantly improved.

ZSQYJDF is composed of multiple components and exhibits the characteristic of acting on multiple targets. Through the integration of multi-source database retrieval and HPLC-Q-Orbitrap-MS analysis, we identified a total of 48 compounds. Among them, 30 components such as protocatechuic acid, emodin, cryptochlorogenic acid, ursolic acid, and quercetin have been confirmed by previous studies to possess significant antioxidant and anti-inflammatory activities (34–37), which is consistent with the results of this study, suggesting that these compounds may be the key material basis for the therapeutic effect of ZSQYJDF. Further network pharmacology analysis (PPI and KEGG enrichment analysis) showed that key targets such as AKT, IL1, TNF, NRF2, and IL-6, as well as antioxidant pathways, play important roles in the pathological process of EMs. Notably, our bioinformatics analysis predicted a synergistic regulatory network composed of multiple signaling pathways (including NF-κB, HIF-1, TNF, etc.), which provides in-depth insights into explaining the overall therapeutic effect of ZSQYJDF with “multi-component, multi-target, and multi-pathway” characteristics.

To further clarify the therapeutic mechanism of ZSQYJDF, we systematically evaluated its efficacy through multiple indicators. ELISA results showed that this formula can significantly reduce the levels of pro-inflammatory factors TNF-α, IL-6, and IL-1β in the serum of EMs rats, confirming its definite anti-inflammatory effect. Histopathological analysis revealed that ZSQYJDF can effectively improve the pathological structure of ectopic endometrium. Based on these findings, we suggest that ZSQYJDF may achieve a comprehensive therapeutic effect on EMs through multiple pathways such as inhibiting inflammatory response and improving the pathological state of the endometrium.

OS (38) is a defensive response of cells to excessive accumulation of ROS and also an important intracellular signal regulation system (30). When the level of ROS exceeds the physiological threshold, it will cause damage to biological macromolecules and simultaneously activate pathways such as Nrf2, inducing the expression of antioxidant enzymes such as SOD and GPx to maintain redox homeostasis (31). Moderate OS can promote cell adaptation, while excessive activation leads to cell damage or death (39). Recent studies have shown that OS is deeply involved in the occurrence and development of EMs, and can promote the proliferation and invasion ability of ectopic endometrial cells (40–42), playing a key regulatory role in its pathological process. Some studies have indicated that EMs can be significantly alleviated by regulating the antioxidant enzyme system and reducing the OS state through the NRF2 signaling pathway (43, 44). The latest research further found that Chinese herbal compounds can reduce EMs lesions by activating the SOD/GPx antioxidant system and inhibiting ROS-mediated inflammatory response (45).

To investigate the effect of ZSQYJDF on OS, we measured the levels of 8-epi-PGF2α, SOD, GSH-PX, and MDA in rat serum, as well as the protein and mRNA expression of NRF2, HO1, NQO1, and KEAP1 in ectopic uterine tissues. The serological results showed significantly elevated levels of oxidative markers (MDA and 8-epi-PGF2α) and significantly downregulated antioxidant indicators (SOD and GSH-PX) in the model group. However, after treatment with ZSQYJDF and mifepristone, the oxidative indicators were significantly reduced, and the antioxidant indicators markedly recovered. At the molecular level, the protein expression of NRF2, HO1, and NQO1 was downregulated, and KEAP1 expression was upregulated in the model group; the ZSQYJDF group showed the opposite trend, with significantly increased expression of NRF2, HO1, and NQO1 and decreased expression of KEAP1. NRF2 is a key transcription factor regulating the antioxidant response, and its inactivation severely impairs the cell’s antioxidant defense capacity (46). Literature shows that As a core regulatory protein in the OS response, NRF2 initiates the transcription of various antioxidant and Phase II detoxifying enzymes, such as HO-1 and NQO1, by binding to the antioxidant response element (ARE), thereby efficiently clearing excess ROS and maintaining intracellular redox balance (47). Impaired function of the NRF2 signaling pathway leads to a significant decrease in cellular antioxidant capacity and subsequent accumulation of oxidative damage (48).

In the pathological process of EMs, inflammation and OS form a vicious cycle, jointly driving disease progression (49). The core mechanism involves activated immune cells (e.g., macrophages) generating large amounts of ROS through NADPH oxidase and mitochondrial dysfunction. ROS act as key signaling molecules that activate the NF-κB and NLRP3 inflammasome pathways, thereby promoting the release of pro-inflammatory factors, such as TNF-α and IL-6. These inflammatory factors can, in turn, stimulate ROS production, forming a self-amplifying positive feedback loop (50). In the EMs microenvironment, this interaction leads to: 1) enhanced proliferation and invasion capabilities of ectopic endometrial cells; 2) accelerated angiogenesis; and 3) aggravated local fibrosis. This study confirms that activation of the NRF2/HO-1 pathway is key to breaking this vicious cycle. Based on this, we propose an integrated mechanistic hypothesis: the multiple active components of ZSQYJDF can converge to activate NRF2, a core signaling hub, thereby orchestrating a synergistic therapeutic network. This network not only directly enhances cellular antioxidant capacity but also, likely through the well-documented mutual antagonism between the NRF2 and NF-κB pathways, mediates the observed downregulation of pro-inflammatory factors (TNF-α, IL-6), thus effectively breaking the interactive vicious cycle of inflammation-OS. Furthermore, the potential crosstalk between NRF2 and HIF-1α signaling in the hypoxic microenvironment of EMs lesions may be another mechanism through which ZSQYJDF inhibits lesion survival and angiogenesis.

**Figure 14:**
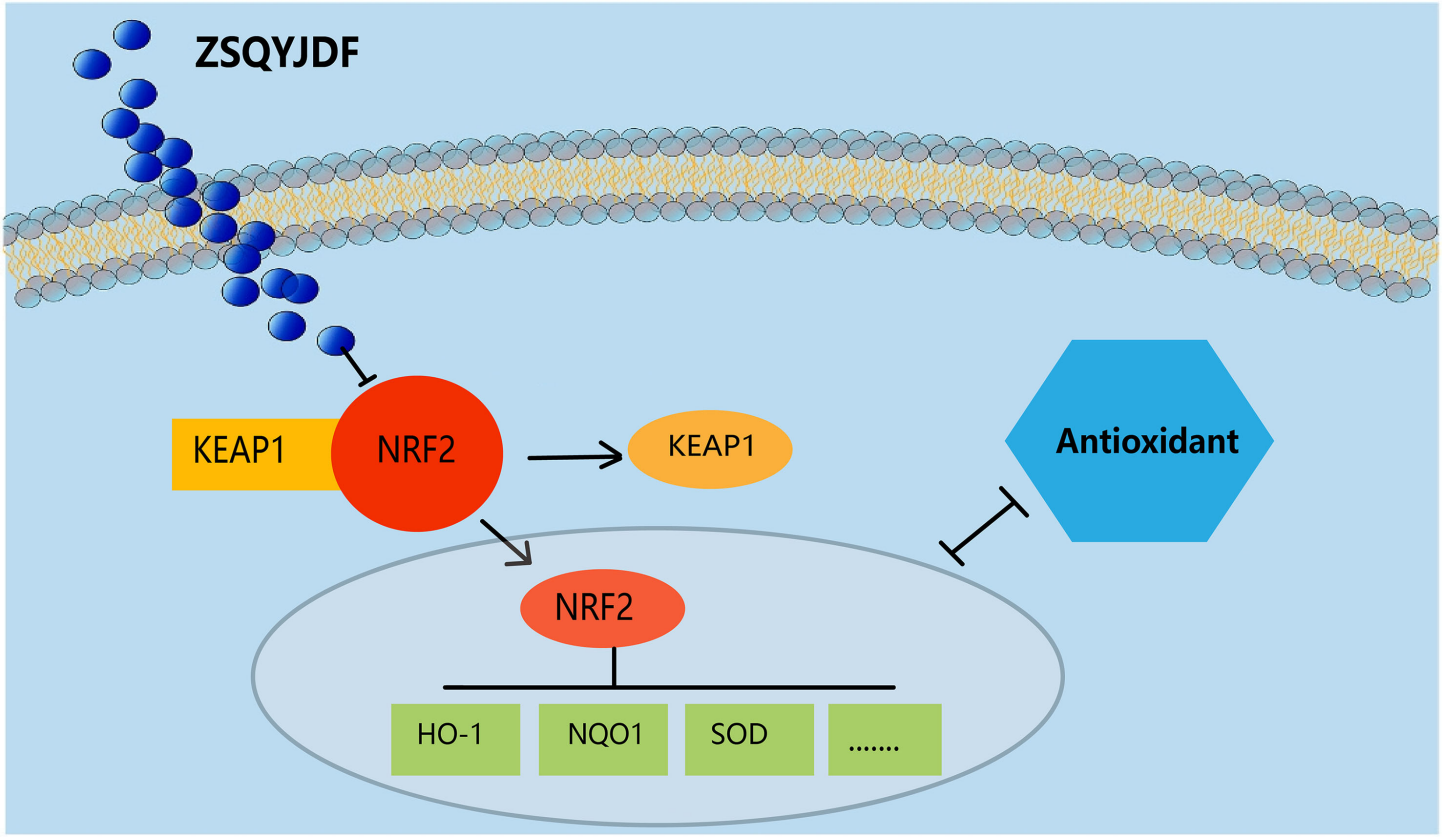
NRF2/HO-1 signaling pathway.

We speculate that after the active components of ZSQYJDF enter the body, they can specifically bind to Nrf2, promoting its dissociation from Keap1 and activating it (**Figure 14**). Activated Nrf2 translocates to the nucleus, initiating the transcription of downstream antioxidant genes (e.g., HO1 and NQO1) while downregulating OS markers (e.g., MDA and 8-iso-PGF2α) and inflammatory mediators. This series of reactions collectively enhances the body’s antioxidant defense capability, alleviates OS damage to the endometrium, and inhibits inflammatory responses and abnormal cell proliferation/migration, thereby interfering with the development of ectopic lesions in the rat model of EM and ultimately inhibiting their growth. Therefore, this study not only verifies that ZSQYJDF can synergistically regulate key effector molecules in the Nrf2-HO1 pathway through its multi-component composition but also provides an in-depth analysis of the inflammation-OS interaction network, offering new strategies and an experimental basis for the precise treatment of EMs.It should be noted that this study observed that ZSQYJDF upregulates the expression of NRF2, HO-1, and NQO1 in ectopic endometrium, preliminarily suggesting its potential activation of the NRF2 pathway. However, the current conclusion is based solely on molecular expression correlations and has not been confirmed through functional validation to establish a causal relationship between NRF2 activation and the therapeutic efficacy of ZSQYJDF. Furthermore, while this study focused on the core role of the NRF2/HO-1 axis, it did not experimentally validate other predicted pathways such as NF-κB and HIF-1α, which somewhat limits a comprehensive understanding of the entire network mechanism. These represent the main limitations of this research. Subsequent studies will employ functional approaches to clarify the functional role of NRF2 and will incorporate pathway-specific inhibitors or genetic manipulation techniques to dissect the contributions and interactions of various pathways within the synergistic network, thereby addressing the current shortcomings.Despite these limitations, this study still holds significant academic value. On one hand, it establishes for the first time an evidence chain linking ZSQYJDF to the NRF2 pathway. On the other hand, it also lays a methodological foundation and experimental framework for future in-depth research on the regulation of the NRF2 pathway by traditional Chinese medicine compound formulations.

## 5 Conclusion

In summary, this study integrated network pharmacology and HPLC-Q-Orbitrap-MS chemical analyses to identify the active components of ZSQYJDF. Combined with *in vivo* experimental validation, it was revealed that the mechanism by which ZSQYJDF treats EMs is likely closely related to the activation of the NRF2/HO1 signaling pathway. This study deepens our understanding of the complex mechanisms of action of Chinese herbal formulas and provides a solid theoretical and experimental foundation for the clinical development of innovative drugs for the treatment of EMs.

## Data Availability

The original contributions presented in the study are included in the article/supplementary material. Further inquiries can be directed to the corresponding author.
